# Identification of genes for salt tolerance and yield-related traits in rice plants grown hydroponically and under saline field conditions by genome-wide association study

**DOI:** 10.1186/s12284-019-0349-z

**Published:** 2019-12-02

**Authors:** Chen Liu, Kai Chen, Xiuqin Zhao, Xiaoqian Wang, Congcong Shen, Yajun Zhu, Mingli Dai, Xianjin Qiu, Rongwei Yang, Danying Xing, Yunlong Pang, Jianlong Xu

**Affiliations:** 1 Present Address: College of Agriculture, Yangtze University, Jingzhou, 434025 China; 2 Present Address: Laboratory of Lingnan Modern Agriculture/ Agricultural Genomics Institute at Shenzhen, Shenzhen, 518120 China; 3 Present Address: Institute of Crop Sciences, Chinese Academy of Agricultural Sciences, Beijing, 100081 China; 4 State Key Laboratory of Crop Biology, College of Agronomy, Shandong Agricultural University, Taian, 271018 China

**Keywords:** Rice, Salt tolerance, Genome-wide association study (GWAS), Candidate gene, Quantitative trait locus/loci (QTL)

## Abstract

**Background:**

Soil salinity is one of the main environmental conditions that affects rice production. Identifying the genetic loci that affect rice salt tolerance (ST)-related traits at the seedling stage, especially under saline field conditions, is crucial for ST rice breeding by pyramiding ST genes that act at different developmental stages.

**Results:**

Large phenotypic variations were observed in 708 rice accessions, and yield and its related traits were considerably limited when exposed to salt stress. In a genome-wide association study (GWAS), 2255 marker-trait association signals were detected for all measured traits, and the significant SNPs were distributed in 903 genes. Of these, 43 genes processed same functional annotation, and the gene ontology terms “biological processes” and “molecular function” with the known genes responsive to salt stress in rice. Further haplotype analysis detected 15 promising candidates significantly associated with the target traits, including five known genes and 10 novel genes. We identified seven accessions carrying favorable haplotypes of four genes significantly associated with grain yield that performed well under saline stress conditions.

**Conclusions:**

Using high density SNPs within genes to conduct GWAS is an effective way to identify candidate genes for salt tolerance in rice. Five known genes (*OsMYB6*, *OsGAMYB, OsHKT1;4*, *OsCTR3*, and *OsSUT1*) and two newly identified genes (*LOC_Os02g49700*, *LOC_Os03g28300*) significantly associated with grain yield and its related traits under saline stress conditions were identified. These promising candidates provide valuable resources for validating potential ST-related genes and will facilitate rice breeding for salt tolerance through marker-assisted selection.

## Background

Rice (*Oryza sativa* L.), one of the most important crops, is a staple food in the diet of more than half of the world’s population. Rice production is adversely influenced by numerous biotic and abiotic stresses including diseases and pests, cold, drought, flooding, heat, and salinity. Soil salinity is a common abiotic stress in many rice production areas that causes serious reductions in productivity. Globally, more than 930 million ha of land are thought to be affected by salinity, with China having over 100 million ha (Hu et al. 2012). Due to current salinity levels and other soil-related problems, millions of hectares in the humid regions of South and Southeast Asia where rice is the main food crop are either left uncultivated, or rice is grown with extremely low yields. Increasing soil salinization is one of the main obstacles affecting rice production. Developing rice varieties with good salt tolerance (ST) and high yield is considered to be one of the most economic options to utilize salinized soil for rice production (Blumwald and Grover 2006).

Generally, rice is considered to be moderately sensitive to salinity (Ismail and Thomson 2011). When the electrical conductivity of soil saturated extract is 5–6 ds/m, the growth of salt-sensitive rice would be negatively affected and the yield would be reduced by half (Abdullah et al. 2001; Fageria 1985; Yeo et al. 1990). However, different rice genotypes vary considerably in their ST characteristics (Gregorio et al. 2002), which provides the basis of breeding for ST rice varieties. To decipher the genetic architecture of ST in rice, the quantitative trait loci (QTL) mapping approach has been used by many researchers to identify QTL at various growth stages including germination (Cheng et al. 2015; Wang et al. 2011), seedling (Cheng et al. 2011; Wang et al. 2012; Zheng et al. 2015) and the reproductive stages (Chai et al. 2013; Hossain et al. 2015; Kumar et al. 2015).

The seedling stage is one of the most vulnerable growth stages to salt stress; tolerance can be measured fairly easily, and many genes affecting ST in seedlings have been identified. *SKC1* was the first ST-related gene to be cloned in rice via map-based cloning (Ren et al. 2005). Through the use of mutants and gene overexpression and knockout, approximately 200 genes have been shown to be involved in salt stress at the seedling stage (Molla et al. 2015). ST at the reproductive stage is necessary for high yield under saline conditions. A large number of QTL for ST-related traits at the reproductive stage have been identified through linkage mapping and association analysis. For example, in an F_2_ population derived from a cross between ‘Cheriviruppu’ (salt-tolerant) and ‘Pusa Basmati 1’ (salt-sensitive), Hossain et al. (2015) detected 16 QTL affecting plant height, tiller number, panicle length, grain yield per plant, biomass, pollen fertility, and Na^+^ content and the K^+^/Na^+^ ratio in the flag leaf measured under stress conditions at the reproductive stage. Kumar et al. (2015) conducted an association study using 220 *Xian* (*indica*) lines genotyped using a custom-designed 6 K single nucleotide polymorphisms (SNPs) array and identified 64 SNPs significantly associated with the K^+^/Na^+^ ratio as well as other agronomic traits including filled grain number, productive tiller number, seed setting rate, stress susceptibility index of the seed setting rate, and grain yield observed at the reproductive stage under salt stress. However, to the best of our knowledge, no QTL for ST at the reproductive stage have been fine-mapped or cloned.

Over the past several years, association studies using high density genome-wide SNP markers detected by next-generation sequencing have been shown to be a powerful strategy to mine candidate genes associated with traits of interest. Recently, the 3000 rice genomes project (3 K RGP) used Illumina next-generation sequencing of a core collection of 3024 rice accessions from 89 countries to generate sequence data with high coverage (~ 94%) and mapping rate (~ 92.5%) and to construct a high-density SNP database providing genotypic data for genome-wide association studies (GWAS) of agronomic traits in rice (Wang et al. 2018).

In this study, 708 rice accessions selected from the 3024 rice genomes sequenced by the 3 K RGP were used to conduct an association study to identify genetic loci for ST. For this analysis, we used 1,101,404 SNPs within genes filtered from the 3 K RGP 32 M SNP raw dataset in the Rice SNP-Seek Database (Alexandrov et al. 2015). The goals of this study were to: 1) identify genes associated with rice ST at the seedling stage, 2) detect genes associated with yield and its related traits under saline field conditions, and 3) analyze promising gene candidates that are potentially involved in tolerance to salt stress and identify favorable haplotypes. This information will be useful for further mining of functional genes for rice ST, and for the marker-assisted breeding of ST varieties suitable for cultivation in salinized paddies.

## Materials and methods

### Plant materials

In total, 708 rice accessions selected from the 3 K RGP were used in this study, which included 400 *Xian* (*indica*), 247 *Geng* (*japonica*), 39 intermediate types, 16 Aus, and six Basmati varieties (Wang et al. 2018). The geographical origins of these accessions included 77 countries or regions with most of accessions originating from China (205), followed by the Philippines (70), India (56), Japan (38), Italy (25), and the United States (24). For other countries or regions, the number of accessions were < 20 (Additional file 1: Table S1).

### Evaluation of ST at the seedling stage under hydroponic conditions

An experiment to evaluate ST at the seedling stage was carried out in a greenhouse at the Institute of Crop Sciences, Chinese Academy of Agricultural Sciences (CAAS), Beijing. Plastic containers were prepared for the screening, and styrofoam seedling float trays were used that fit inside the plastic containers. Each styrofoam tray consists of an array of 10 × 13 holes with a styrofoam nylon net attached to the bottom. The rice seeds were surface sterilized with 5% sodium hypochlorite solution for 20 min and rinsed well with distilled water. Seeds were soaked in water for 48 h at 30 °C to allow them to germinate. A single healthy germinated seed was placed in each hole, and 10 seeds were sown for each accession. During the first two days, the seedlings were watered with tap water, after which it was changed to Yoshida culture solution (Yoshida et al. 1976). When the seedlings were at the three-leaf stage two weeks after seeding, NaCl was added to the culture solution at concentrations up to 140 mM. The pH of the culture solution was adjusted daily to 5.0 by adding either NaOH or HCl, and the solution was renewed every five days. The seedlings were scored based on visual symptoms of salt stress injury (SSI) when the susceptible check IR29 was given a score of 9 as described in the modified Standard Evaluation System (IRRI 2013). Seedling survival days (SSD) was recorded on a daily basis after the first plant had died.

### Yield trials under saline field conditions

The association panel was evaluated for grain yield and related traits in replicated field trials conducted under salt stress condition near the coastline at Sanmen (18.3°N, 109.3°E) in Zhejiang Province, China in 2016 (Y16) and 2017 (Y17). Field trials were conducted following a randomized complete block design with two replicates in 2016 and three replicates in 2017. Twenty germinated seeds were directly and evenly sown in a non-salt-stress normal field in 50 cm-long rows with 25 cm spacing between rows. Salt stress was introduced after the 2-leaf stage and maintained up to maturity by irrigating with 0.5% salt water made by proportionally mixing river and sea water. Seedling numbers (SN) in each plot were counted one month after salt application. Heading date (HD, in days) was visually recorded in Y17 for each plot when ~ 50% of plants in a plot had headed. At maturity, total panicle number (PN) was recorded and five uniform main panicles in the middle of each plot were sampled to measure total spikelet number (TSN), filled grain number (FGN), seed setting rate (SSR, %), and thousand-grain weight (TGW, in g). The remaining seeds in each plot were harvested and bulked to measure grain yield per plot (GY, in g).

### Statistical phenotypic analyses

Phenotypic analyses were conducted using linear mixed models. For single-site analysis, accession (genotype) was regarded as a fixed effect and replicate as random effect. The best linear unbiased estimates (BLUE) of the accessions were obtained. For multi-site analysis, all effects including accession (genotype), environment, and replicate within environment were regarded as random to estimate variance components. Narrow-sense heritability (h^2^) based on genotypic means was computed using the estimated variance components as V_G_/(V_G_ + V_GEI_/s + V_e_/sr). Where V_G_, V_GEI_, and V_e_ are the variance due to genotype, genotype by environment interaction (GEI), and residual error, respectively; s is the number of environments and r is the number of replicates. All analyses were conducted using the PBTools package developed by IRRI (bbi.irri.org). Phenotypic correlations were computed using the BLUE with the “rcorr” function implemented in the R package Hmisc (Harrell 2015).

### Manipulating genotype data from the 3 K RGP

The 32 M SNPs raw data of the association panel were extracted from the 3 K RGP in the Rice SNP-Seek Database (http://snp-seek.irri.org/). Genotype cleaning was carried out according to the following steps: 1) for SNPs with more than two allele types, only the top two major alleles were retained, and the other allele types were regarded as missing data; 2) SNPs with minor allele frequency (MAF) < 0.05 and missing rate > 20% were removed; 3) a subset of SNPs were randomly sampled for analyzing population structure by calculating kinship and performing a principle component analysis (PCA) using the GAPIT R package (version 3.0); 4) based on gene functional annotations of the reference *Geng* (*japonica*) ‘Nipponbare’ genome IRGSP-1.0 from the Rice Genome Annotation Project (http://rice.plantbiology.msu.edu/), SNPs within annotated genes were extracted to perform a GWAS.

### GWAS using gene-based SNPs

We carried out GWAS to detect marker-trait associations for all measured traits using SNPs within annotated genes using the GAPIT (version 3.0) R package that uses EMMA (efficient mixed model association), CMLM (compressed mixed linear model), and P3D (population parameters previously determined) to speed up computation time and optimize statistical performance in the GWAS. The mixed linear model (MLM) allowed correction for cryptic relatedness and other fixed effects using kinship matrix (K) and population stratification through principle components (P). The Bonferroni multiple testing correction was applied to identify significant markers. Significant SNPs affecting the investigated traits were claimed when the test statistics reached *P* < 1.0 × 10^− 4^. Individual marker effects were extracted from the model and visualized in a Manhattan plot prepared with the R package qqman (Turner 2014).

### Identifying promising candidate genes for salt tolerance

To detect promising candidate genes for ST in rice, the identified associated genes were compared with genes that are known to be responsive to salt stress from searches of the funRiceGenes (https://funricegenes.github.io/) and QTARO databases (http://qtaro.abr.affrc.go.jp). Gene ontology (GO) analysis was carried out using ‘AgriGO v2’ (http://bioinfo.cau.edu.cn/agriGO/analysis.php). Gene functional annotations were extracted from the MSU Rice Genome Annotation Project Release 7. For genes showing the same functional annotation and GO terms with known genes, haplotype analysis was further carried out and the differences in target traits between/among major haplotypes (with more than 10 accessions) were tested by ANOVA. The genes with significance level *P* < 0.001 were considered to be promising ST candidates.

## Results

### Phenotypic variation and correlations

**Fig. 1.**
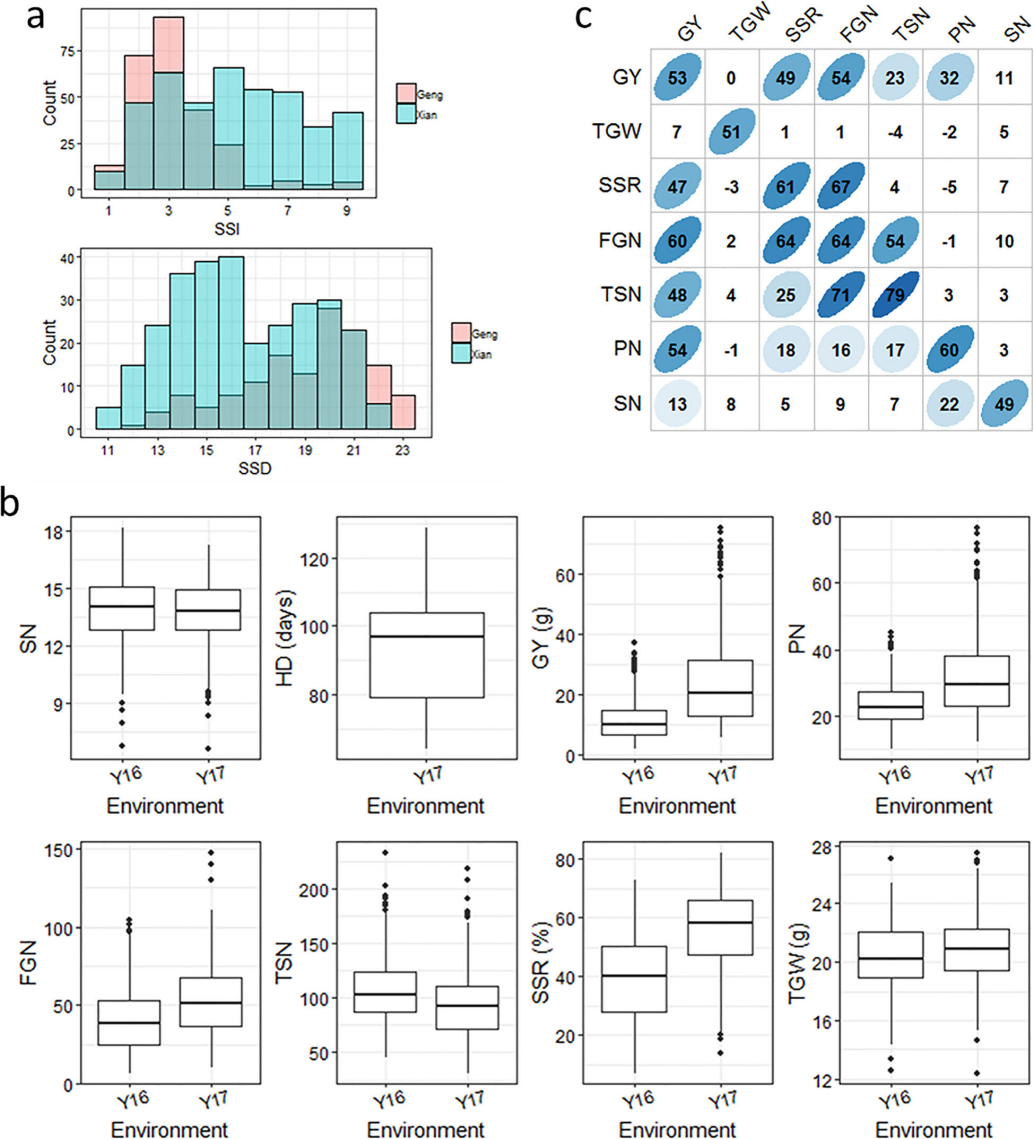
Phenotypic distributions and correlations. (a) Distributions of SSI (salt stress injury) and SSD (seedling survival days) measured under hydroponic condition. “Count” indicated the number of rice accessions. Blue and red indicated *Xian* (*Indica*) and *Geng* (*Japonica*) subspecies, respectively. (b) Distributions of traits measured under saline field conditions. SN: seedling number; HD: heading date; GY: grain yield per plot; PN: total panicle number; FGN: filled grain number; TGW: thousand-grain weight; TSN: total spikelet number per plot; SSR: seed setting rate. (c) Pairwise correlations of traits measured under saline field conditions. The correlation coefficients (r) multiplied by 100 on the lower triangle, upper triangle, and principal diagonal show the correlations in 2016, 2017, and between the two years, respectively. The areas and colors of the ellipses show the absolute values of the corresponding r. Right and left oblique ellipses indicate positive and negative correlations, respectively. The values without ellipses indicate that the correlations were not significant at *P* < 0.05.

ST at the seedling stage varied widely among the accessions, with SSI ranging from 1 to 9 and SSD ranging from 11 to 23 days. Overall, the *Geng* (*japonica*) subspecies had lower values for SSI and higher values for SSD compared with the *Xian* (*indica*) subspecies. The mean SSI (SSD) was 3.3 (19.0) for the *Geng* subspecies but 5.2 (16.5) for the *Xian* subspecies, which indicated that *Geng*-type varieties are more tolerant to salt stress than are *Xian* varieties (Fig. 1 a). Large variations in all the investigated agronomic traits were observed in the rice association panel grown under salt stress conditions in the two field years. Salt stress seriously limits growth and grain yield in rice. The performance in Y16 was slightly worse than it was in Y17, suggesting that salt stress experienced in Y16 was more severe (Fig. 1 b). GY was significantly positively correlated with its components except TGW. The trait correlation coefficients (r) between the two years ranged from 0.51 for TGW to 0.79 for TSN (Fig. 1 c). Variance component analysis of the traits showed that V_G_ was larger than the other components, indicating that the phenotypic variances were mainly determined by genotype. The estimated heritability (*h*^*2*^) varied from 0.59 for TGW to 0.88 for TSN (Table 1).

**Table 1:** Variance components and heritability estimates for agronomic traits investigated under salt stress conditions in 2016 and 2017

Trait	V_G_	V_GEI_	V_Rep_	V_E_	V_e_	*h*^*2*^
GY	109.55	48.88	4.11	99.56	126.49	0.70
FGN	343.18	128.0	19.13	77.41	300.96	0.75
TSN	829.52	54.01	11.39	125.53	512.92	0.88
SSR	147.59	57.66	15.16	149.23	143.26	0.74
TGW	2.63	2.62	0.24	0.04	2.06	0.59
PN	90.69	9.85	6.04	55.18	113.21	0.80
SN	1.60	1.40	0.24	0.00	3.26	0.60

V_G_: genotype variance; V_GEI_: genotype by environment interaction variance; V_E_: environment variance; V_Rep_: replication variance within environment; V_e_: residual variance; *h*^*2*^: narrow-sense heritability. Trait abbreviations are as given in the legend to Figure 1.

### Distribution of SNPs

After removing SNPs with MAF < 0.05 and missing data rate > 20%, there wer 3,455,952 SNPs remaining; the number of SNPs per chromosome ranged from 212,314 (chromosome 9) to 404,439 (chromosome 1). We randomly sampled 10% (345,600) of the SNPs for the kinship and PCA analyses. The average marker spacing over the whole genome was 1079 bp with a range of 933 bp on chromosome 10 to 1244 bp on chromosome 5. In total, we identified 1,101,404 SNPs in 44,332 annotated genes. Each gene contained around 25 SNPs on average ranging from at least one SNP to as many as 649 SNPs (Table 2).

**Table 2:** Distribution of high quality SNP markers in the rice genome

Chr	All SNPs ^a^		10% set ^b^		SNPs within genes ^c^		
	No. of SNPs	Spacing (bp)	No. of SNPs	Spacing (bp)	No. of genes	No. of SNPs	Spacing (bp)
1	404,439	107	40,444	1070	5468	128,891	336
2	337,347	107	33,735	1065	4577	105,849	339
3	311,509	117	31,151	1169	4962	94,538	385
4	298,569	119	29,857	1189	3758	98,508	360
5	240,251	125	24,026	1244	3812	73,139	409
6	290,332	108	29,034	1073	3720	91,561	340
7	275,472	108	27,548	1077	3482	88,151	336
8	281,408	101	28,141	1010	3278	90,526	314
9	212,314	108	21,232	1078	2656	67,287	340
10	248,350	93	24,835	934	2724	77,376	299
11	300,367	97	30,037	965	3088	102,704	282
12	255,594	108	25,560	1077	2807	82,874	332
Total	3,455,952	108	345,600	1079	44,332	1,101,404	339

^a^All SNPs with minor allele frequency > 0.05 and missing data rate < 20%; ^b^Randomly sampled set of 10% of the SNPs from the complete SNP dataset; ^c^All SNPs distributed in 44,332 annotated genes

### GWAS using gene-based SNPs

**Fig. 2:**
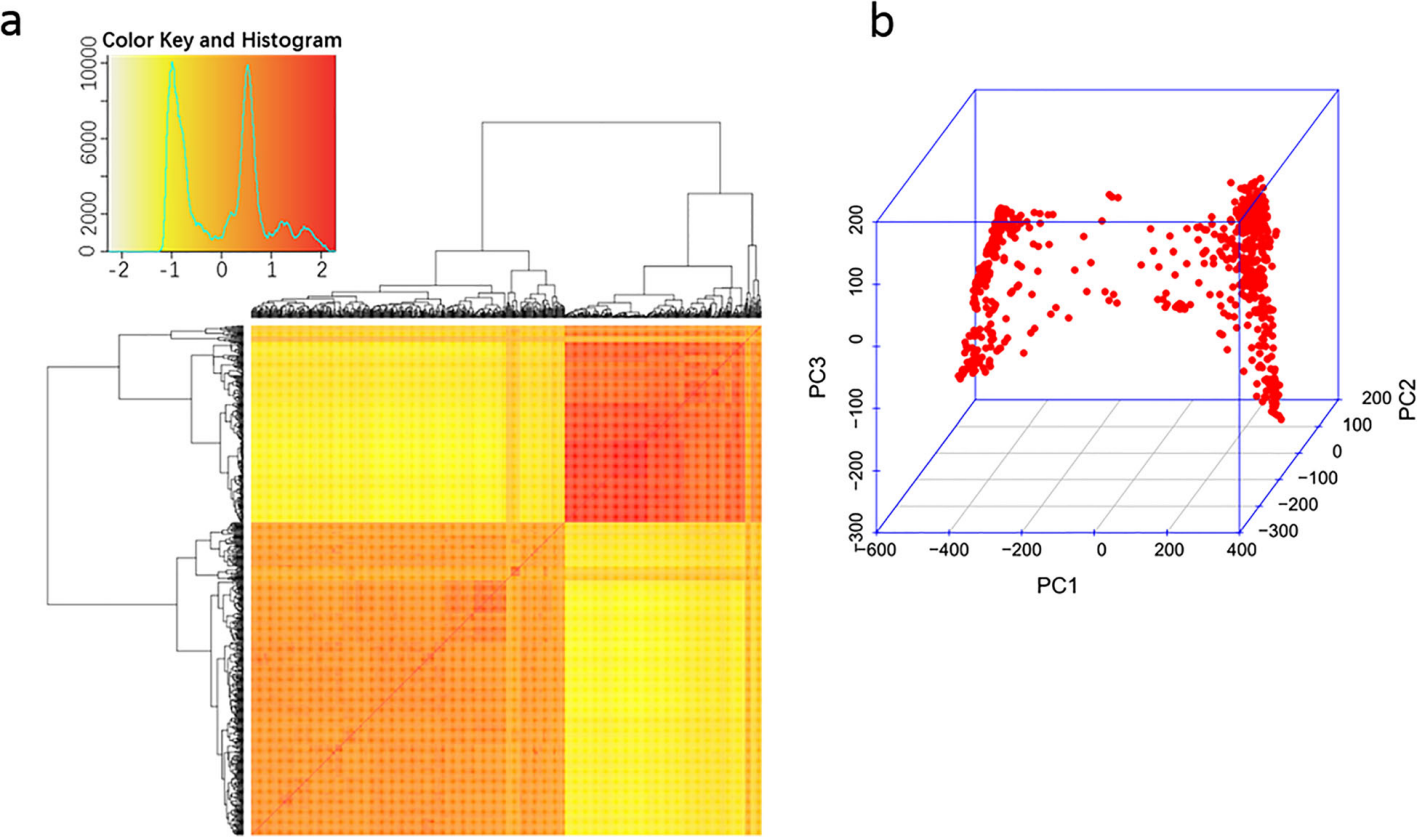
Population structure of the rice germplasm association panel revealed by kinship (a) and principle component analysis (PCA) (b)

The kinship and PCA analyses showed two distinct subpopulations in the association panel (Fig. 2). One group was comprised of 265 accessions mainly belonging to the *Geng* subspecies, and the other group was comprised of 443 accessions mainly belonging to the *Xian* subspecies (Additional file 1: Table S1). Thus, to partially eliminate the false positives induced by population structure, we performed a GWAS for each group separately. The Manhattan plots and QQ plots of the associations for each trait are shown in Additional file 1: Figure. S2A-I.

At the seedling stage, 321 marker-trait associations were detected that included 108 associations identified in the *Geng* group and 213 associations in the *Xian* group, with none of these associations detected in both groups. The SNP markers were distributed in 142 gene regions which included 188 SNPs in 95 genes associated with SSI, and 133 SNPs in 63 genes for SSD. Among them, 49 SNPs in 16 genes were associated with the two traits simultaneously (Table 3 and Additional file 1: Table S2).

**Table 3:** Summary of the GWAS results for all 10 measured traits

Condition	Group	Association	SSI	SSD	SN	HD	GY	PN	TSN	FGN	SSR	TGW	Total
Hydroponics	*Geng*	No. SNPs	83	25	–	–	–	–	–	–	–	–	107
		No. SNPs	56	13	–	–	–	–	–	–	–	–	68
	*Xian*	No. SNPs	105	108	–	–	–	–	–	–	–	–	165
		No. SNPs	39	50	–	–	–	–	–	–	–	–	74
	Total	No. SNPs	188	133	–	–	–	–	–	–	–	–	272
		No. SNPs	95	63	–	–	–	–	–	–	–	–	142
Y16	*Geng*	No. SNPs	–	–	12	–	6	129	7	6	9	0	169
		No. SNPs	–	–	11	–	5	44	7	5	2	0	74
	*Xian*	No. SNPs	–	–	257	–	21	101	18	282	58	179	862
		No. SNPs	–	–	93	–	15	52	15	88	48	63	342
	Total	No. SNPs	–	–	269	–	27	230	25	288	67	179	1031
		No. SNPs	–	–	104	–	20	96	22	93	50	63	415
Y17	*Geng*	No. SNPs	–	–	85	16	113	48	16	59	13	0	341
		No. SNPs	–	–	22	14	58	23	12	22	8	0	151
	*Xian*	No. SNPs	–	–	101	19	19	41	191	61	63	4	492
		No. SNPs	–	–	57	14	10	26	85	46	21	3	256
	Total	No. SNPs	–	–	186	35	132	89	207	120	76	4	832
		No. SNPs	–	–	79	28	68	49	97	68	29	3	403

Under saline field conditions in 2016, 1085 marker-trait associations for all seven measured traits were detected including 169 associations identified in the *Geng* group and 916 identified in the *Xian* group. None of the associations were detected in both groups. These SNP markers were distributed in 415 genes which included 67 SNPs in 50 genes associated with SN, 27 SNPs in 20 genes associated with GY, 230 SNPs in 96 genes associated with PN, 269 SNPs in 104 genes associated with TSN, 288 SNPs in 93 genes associated with FGN, 179 SNPs in 63 genes associated with SSR, and 25 SNPs in 22 genes associated with TGW (Table 3 and Additional file 1: Table S2).

Under saline field conditions in 2017, 849 marker-trait associations for all measured traits were detected including 350 associations identified in the *Geng* group and 499 identified in the *Xian* group. These SNP markers were distributed in 403 genes which included 186 SNPs in 79 genes associated with SN, 35 SNPs in 28 genes associated with HD, 132 SNPs in 68 genes associated with GY, 89 SNPs in 49 genes associated with PN, 207 SNPs in 97 genes associated with TSN, 120 SNPs in 68 genes associated with FGN, 76 SNPs in 29 genes associated with SSR, and four SNPs in three genes associated with TGW (Table 3 and Additional file 1: Table S2).

### Promising candidate genes for ST in rice

**Fig. 3:**
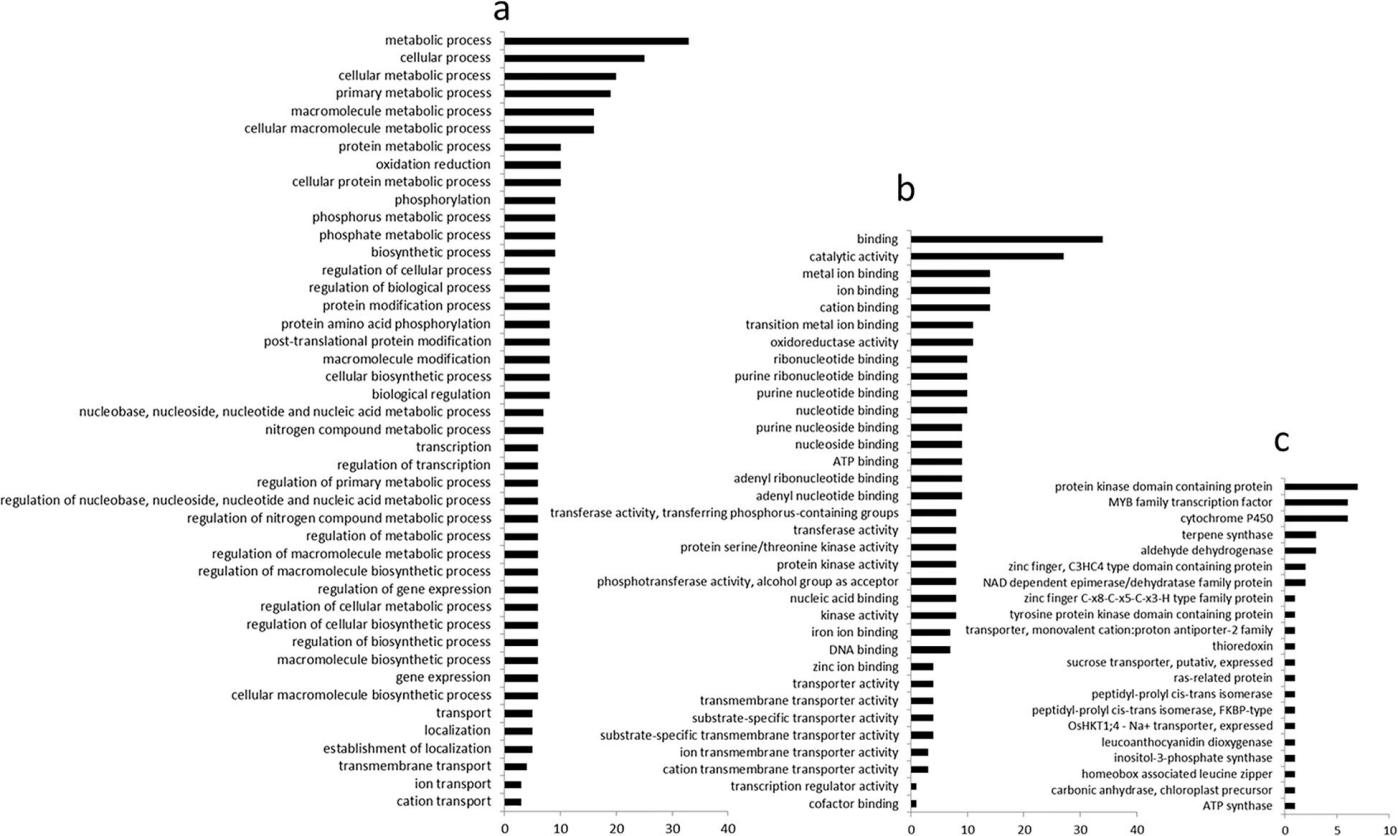
The GO terms represented in the “biological process” (a) and “molecular function” (b) GO categories, and the putative encoded proteins (c) for the 43 identified candidate genes

**Fig. 4:**
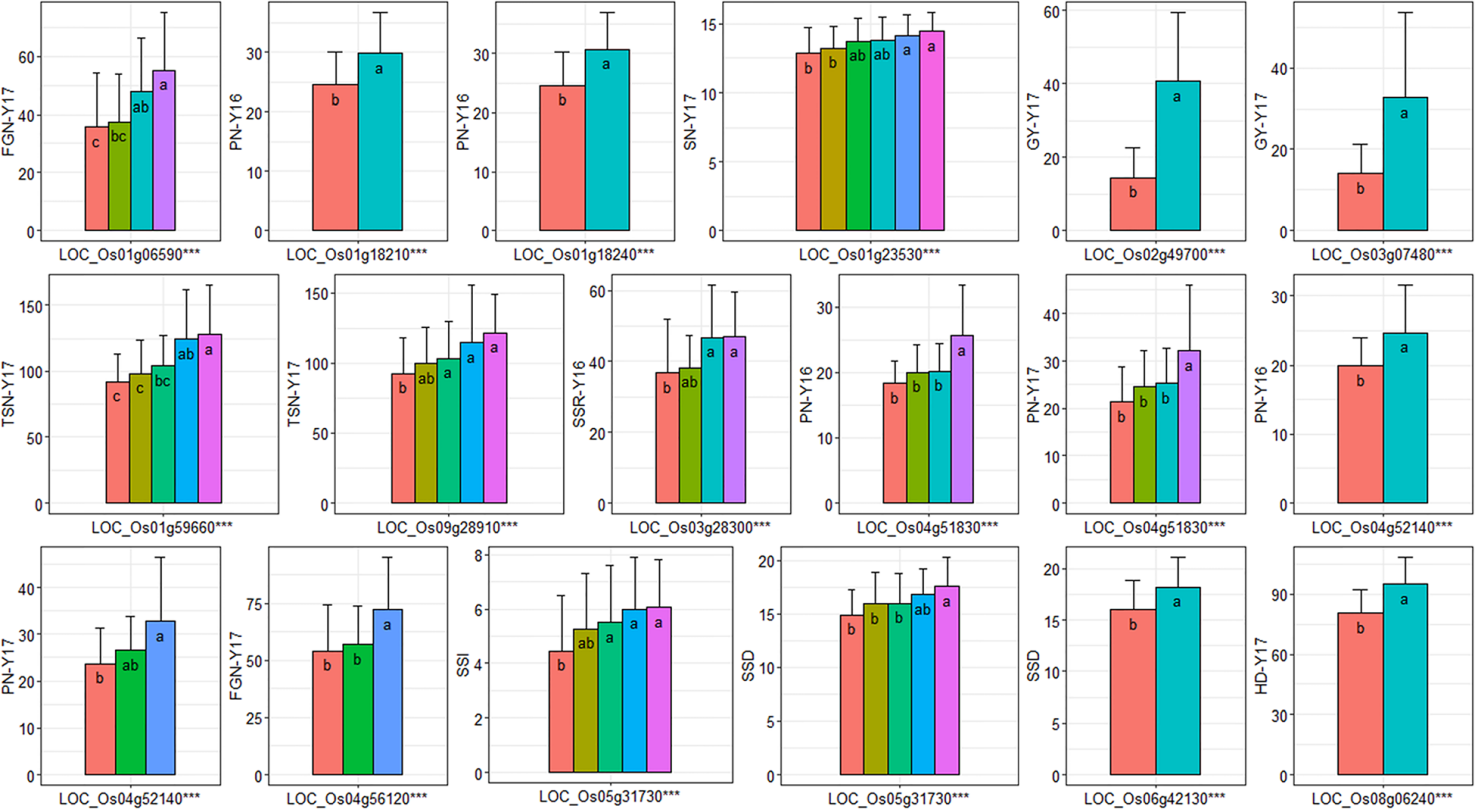
Haplotype analysis of the 15 promising candidate genes for rice salt tolerance. The colors of the bars indicate the different haplotypes for each gene. *** suggested significant differences in the targeted traits among/between haplotypes tested by ANOVA at *P* < 0.001. Lower-case letters in histograms were ranked by Duncan’s test at *P* < 0.05

In total, 230 genes in which expression is known to be responsive to salt stress were identified by searches of the funRiceGenes and QTARO databases (Additional file 1: Table S3). Among the 903 rice genes associated with the measured traits, 43 genes shared the same functional annotations and GO terms in the “biological process” and “molecular function” GO categories with the 230 known genes (Additional file 1: Table S4). These genes encode 21 types of putative proteins involving 35 different molecular functions and 45 biological processes (Fig. 3 and Table 4). We performed haplotype analyses and identified 2–6 major haplotypes (comprising more than 10 accessions) for these 43 genes (Fig. 4). The differences in target traits between/among haplotypes were tested by ANOVA (Additional file 1: Table S5), and 15 significant genes were detected, including one gene for SSI, two genes for SSD, one gene for HD, two genes for GY, four genes for PN, two genes for FGN, one gene for SSR, and two genes for TSN (Table 4 and Fig. 4). Among them, *LOC_Os04g51830* and *LOC_Os04g52140* were detected for PN in both 2016 and 2017, and *LOC_Os05g31730* was detected for SSI and SSD. The above genes encompassed 10 different predicted functions, such as a MYB family transcription factor, protein kinase domain containing proteins, and transporter proteins (Table 4).

**Table 4:** Fifteen promising candidate genes for rice salt tolerance and their associations with the targeted traits identified by haplotype analysis

MSU.ID ^a^	Function	GWAS ^b^		ANOVA ^c^		Trait ^d^	Env ^e^
		*P* value	R^2^(%)	P value	R^2^(%)		
*Os09g28910*	carbonic anhydrase, chloroplast precursor	6.60E-05	3.5	0.0007	5.3	TSN	Y17
*Os02g49700*	homeobox associated leucine zipper	5.20E-07	11.8	1.10E-18	32.7	GY	Y17
*Os06g42130*	leucoanthocyanidin dioxygenase	7.90E-05	5.2	1.30E-05	8.1	SSD	Hydroponics
*Os01g18240*	MYB family transcription factor	4.00E-05	5.1	1.50E-07	8.6	PN	Y16
*Os01g59660*	MYB family transcription factor	7.70E-05	3.5	4.10E-07	10.6	TSN	Y17
*Os08g06240*	MYB family transcription factor	8.30E-05	4.4	4.70E-15	24.6	HD	Y17
*Os04g51830*	OsHKT1;4 - Na + transporter	1.60E-05	10.8	1.90E-05	14.1	PN	Y16
*Os04g51830*	OsHKT1;4 - Na + transporter	1.80E-05	7.6	2.20E-05	10.9	PN	Y17
*Os01g18210*	peptidyl-prolyl cis-trans isomerase, FKBP-type	6.30E-05	4.8	9.10E-06	6.3	PN	Y16
*Os03g28300*	protein kinase domain containing protein	1.40E-05	6	3.90E-06	10.6	SSR	Y16
*Os04g52140*	protein kinase domain containing protein	4.70E-05	9.6	1.40E-06	13.7	PN	Y16
*Os04g52140*	protein kinase domain containing protein	9.70E-05	6.2	2.00E-07	13.3	PN	Y17
*Os04g56120*	protein kinase domain containing protein	8.80E-05	3.8	2.40E-08	9.7	FGN	Y17
*Os03g07480*	sucrose transporter	3.10E-05	8	6.40E-14	26.4	GY	Y17
*Os01g23530*	terpene synthase	5.30E-05	3.7	0.0004	7.1	SN	Y17
*Os05g31730*	transporter, monovalent cation:proton antiporter-2 family	2.10E-07	6.2	5.40E-06	9.7	SSI	Hydroponics
*Os05g31730*	transporter, monovalent cation:proton antiporter-2 family	2.00E-05	6.1	2.90E-05	12.2	SSD	Hydroponics
*Os01g06590*	zinc finger, C3HC4 type domain containing protein	8.20E-05	7.2	4.90E-06	13.4	FGN	Y17

^a^Gene names from the MSU Rice Genome Annotation Project Database; ^b^ GWAS statistics for peak SNP within the gene; ^c^ ANOVA for haplotypes of the gene with target traits; ^d^ For trait name abbreviations see **Fig.**

### Promising candidate genes associated with GY under saline conditions

**Fig. 5:**
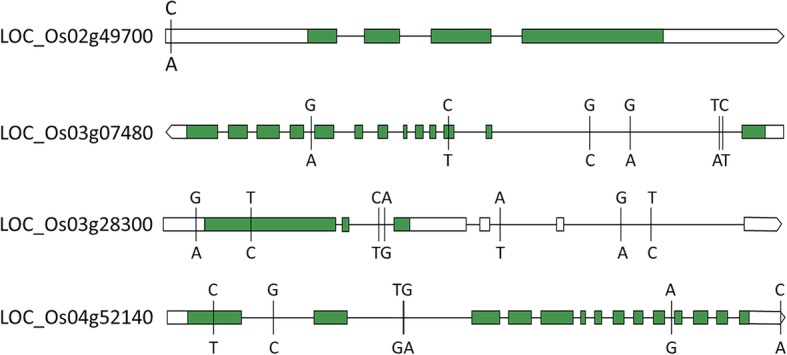
Predicted structures of the four genes significantly associated with GY and the positions of the SNPs used for haplotype analysis. The green rectangles indicated exons

Among the 15 promising candidates, two genes (*LOC_Os02g49700* and *LOC_Os03g07480*) detected in the *Geng* subgroup were especially attractive, because they were associated with GY and explained 32.7% and 26.4% of the phenotypic variance (Table 4). *LOC_Os02g49700* is predicted to encode a homeobox-associated leucine zipper protein. One polymorphic locus, S1_30381323 (A/C), was identified in the 5′ UTR of the gene in the *Geng* subgroup, and the A to C mutation could potentially increase GY significantly under saline stress conditions. For *LOC_Os03g07480*, six polymorphic SNPs were identified in the gene that that accounted for two major haplotypes. All of these SNPs are located in introns except S3_3800635 (T/C) that results in a synonymous mutation (Fig. 5). We further analyzed associations between haplotypes for the other 13 promising genes with GY, and found that another two genes, *LOC_Os03g28300* and *LOC_Os04g52140*, associated with SSR and PN also affected GY and explained 11.5% and 7.7% of the phenotypic variance, respectively, in the *Geng* subgroup (Additional >file 1: Table S6). For *LOC_Os03g28300*, seven SNPs were identified that formed five major haplotypes. One SNP S3_16283872 (T/C) was located in an exon, resulting in a non-synonymous mutation (Ser/Pro), while the other SNPs were in the UTRs and introns (Fig. 5). For *LOC_Os04g52140*, seven polymorphic loci were identified which formed three major haplotypes. All of these SNPs are located in the UTRs or introns except for S4_30974542 (C/T) which is present in an exon and causes an amino acid change from Thr to Met (Fig. 5).

## Discussion

### GWAS using SNPs within genes

GWAS has been widely used in rice for various studies that usually employ tens to hundreds of thousands of SNP markers distributed across the entire genome. Recently, the 3 K RGP sequenced a core collection of 3024 rice accessions and generated more than 32 M SNP data, which provided us the opportunity to perform GWAS using higher density markers (Wang et al. 2018). Also, a GWAS using SNPs within genes allowed us to detect candidate genes directly and reduce computation time as well. In the present study, 3,455,952 high quality SNP markers (MAF ≥0.05 and missing data rate < 20%) were screened from the raw 32 M SNPs. Among these, 1,101,404 SNPs within genes were further extracted to conduct the GWAS which could further reduce computation time dramatically. These SNPs are distributed in 44,332 annotated genes, and cover up to 80% of all annotated genes (55,801) in the MSU Rice Genome Annotation Project Release 7 (http://rice.plantbiology.msu.edu/); this means that most of the genes, if not all, that contribute to the phenotypic variation in the measured traits could be detected. Our study provides a successful example for efficiently using SNP data from the 3 K RGP in a GWAS.

### Promising candidate genes for ST in rice

At present, many genes related to rice ST have been identified by various methods such as map-based cloning, mutation, overexpression, and gene knockout, which allowed us to compare the association mapping results from the present study with previously identified genes. Among the 903 genes associated with SSI and SSD at the seedling stage, and yield and its related traits measured under saline field conditions in Y16 and Y17, 43 candidate genes were identified that shared the same functional annotations and the same GO terms in the “biological process” and “molecular function” GO categories. Of these, 15 genes were further investigated by haplotype analysis and ANOVA. These genes are considered to be potential candidates to improve ST in rice.

Three genes predicted to encode protein kinase domain-containing proteins were detected; *LOC_Os03g28300*, *LOC_Os04g52140*, and *LOC_Os04g56120*. *LOC_Os04g52140* (*OsCTR3*) is predicted to be a Raf-like Ser/Thr protein kinase CTR (constitutive triple-response)-like gene related to ethylene signaling (Wang et al. 2013). Its function with respect to ST in rice has not been reported, but ethylene signaling could modulate the salt response at different levels, including membrane receptors, components in the cytoplasm, and nuclear transcription factors in the pathway (Cao et al. 2008). Here, we identified its association with PN under salt stress conditions. The other two genes are novel genes that have not been reported previously.

Genes encoding MYB-type transcription factors have been shown to play important roles in abiotic stresses in many plant species, including drought and salinity stresses in rice; examples are *OsMYB2* (Yang et al. 2012), *OsJAmyb* (Yokotani et al. 2013), *OsMYB48–1* (Xiong et al. 2014), and *OsMYB91* (Zhu et al. 2015). In the present study, three MYB family transcription factor genes were identified, including *LOC_Os01g18240*, *LOC_Os01g59660*, and *LOC_Os08g06240*. Among these, *LOC_Os01g18240*, also known as *OsMYB61*, regulates cellulose synthase genes (CESAs) in rice (Huang et al. 2015), and *LOC_Os01g59660* (*OsMYBGA*) functions in the induction of gibberellin (GA)-dependent α-amylase in the aleurone and floral organ and pollen development in rice (Kaneko et al. 2004), but the roles these genes play in rice ST was not characterized. The other MYB family transcription factor gene, *LOC_Os08g06240*, that is potentially related to salt stress was first identified here.

*LOC_Os02g49700*, a gene that is associated with GY, encodes a homeobox-associated leucine zipper protein and is the same as the two known genes *OsHOX24* and *Oshox22*. Overexpression of *OsHOX24* enhanced susceptibility to abiotic stresses in transgenic rice by modulating stress-responsive gene expression. The expression of *Oshox22* is strongly induced by salt stress. Rice plants homozygous for a T-DNA insertion in the promoter region of *Oshox22* showed reduced *Oshox22* expression and enhanced tolerance to drought and salt stress at the seedling stage. In contrast, transgenic rice plants over-expressing *Oshox22* showed decreased tolerance to drought and salt (Zhang et al. 2012). The SNPs present in the 5′ UTR of the gene may regulate its expression levels, which could result in increased GY under salt stress conditions.

*LOC_Os03g07480* is a sucrose transporter gene also known as *OsSUT1*. *OsSUT1* plays a role in carbon partitioning, specifically in grain filling and seed germination, and anti-sense lines in which this gene is silenced shown increased tolerance to salt stress (Siahpoosh et al. 2012). An association of *LOC_Os03g07480* with GY under saline stress conditions was identified in our study. We detected five mutations in introns and one synonymous mutation in an exon in this gene; thus, we can speculate that alternative splicing could result from these mutations and possibly alter the gene’s function.

We identified *LOC_Os01g06590*, a gene encoding a zinc finger, C3HC4-type domain-containing protein that is associated with FGN. The functional annotation was the same as that of two previously-identified ST-related genes, *Osdsg1* and *OsMAR1*; the proteins encoded by these genes have been shown to possess E3 ubiquitin ligase activity (Park et al. 2010; Park et al. 2017).

The gene *LOC_Os01g18210* associated with PN encodes a peptidyl-prolyl cis-trans isomerase, the same as two known ST-related genes *OsCYP2* and *OsCYP21–4* that encode cyclophilin proteins. OsCYP2 acts as a key regulator that controls ROS levels by modulating the activities of antioxidant enzymes at the level of translation, and expression of *OsCYP2* is induced by salt stress (Ruan et al. 2011). Transgenic plants overexpressing *OsCYP21–4* exhibited increased tolerance to salinity, possibly via regulation of peroxidase activity (Lee et al. 2015).

We found that *LOC_Os01g23530* was significantly associated with SN. Because the seeds were directly sown in the normal paddy field, the number of surviving seedlings observed one month after salinization from the two-leaf stage could reflect the salt tolerance at the seedling stage. *LOC_Os01g23530* encodes a terpene synthase that is the same as the known ST-related gene *OsKS2*. OsKD2 is an ent-kaurene synthase that participates in GA biosynthesis and plays a significant role in the growth and development of higher plants. Moreover, *OsKS2* transcripts are strongly induced by salinity and drought, which suggests that its expression might be related to abiotic stress defense in rice (Ji et al. 2014).

*LOC_Os04g51830*, also known as *OsHKT1;4*, encodes a Na^+^ transporter. Certain class I transporters of the high-affinity K^+^ transporter (HKT) family have been demonstrated to mediate leaf-blade Na^+^ exclusion during salt stress via Na^+^-selective transport. Suzuki et al. (2016) reported that *OsHKT1;4*-mediated Na^+^ transport in stems contributes to Na^+^ exclusion from rice leaf blades at the reproductive growth stage under salt stress. We detected the association of *LOC_Os04g51830* with PN (Table 4).

*LOC_Os05g31730*, a gene associated with SSI and SSD (Table 4), encodes a transporter belonging to the monovalent cation:proton antiporter-2 family and is the same as two known genes, *OsNHX1* and *OsNHX5*. Proteins encoded by members of this gene family play important roles in the compartmentalization of Na^+^ and K^+^ that accumulate in the cytoplasm into vacuoles, and thus could improve rice ST (Fukuda et al. 2011). *LOC_Os06g42130* encodes leucoanthocyanidin dioxygenase, which is the same as *OsAns*, a rice flavonoid pathway gene that is induced by high salt treatment (Ithal and Reddy 2004). *LOC_Os06g42130* was found to be associated with SSD. *LOC_Os09g28910* is associated with TSN and encodes carbonic anhydrase (CA), and the chloroplast precursor is same as that encoded by *OsCA1*. The expression of *OsCA1* in rice leaves and roots is induced by salt stress (Yu et al. 2007).

### Application in breeding rice for ST

Rice plants may be subject to salt stress at any development stage and even over the entire growth period. Improving yield under saline stress conditions is the final goal of rice ST breeding. Because the correlation between the physiological indicators at the seedling stage and final yield at the reproductive stage under salt stress is poor (Hossain et al., 2015), we also found that rice plants that are salt tolerant as seedlings might not necessarily be high yielding when grown in a salinized paddy field, indicating that the genetic basis of rice ST at the seedling and reproductive stages is different. Interestingly, in the current study, we still identified 10 genes associated with both ST-related traits (SSI, SSD, and/or SN) at the seedling stage and yield and its related traits (GY, PN, TSN, FGN, SSR, and/or TGW) (Additional file 1: Table S2), which might be due to pleiotropism or linkage disequilibrium. Thus, pyramiding genes that function at different developmental stages or all stages will facilitate molecular breeding for ST in rice. Of the 15 promising genes for rice ST identified here, four genes (*LOC_Os02g49700*, *LOC_Os03g28300*, *OsCTR3*, and *OsSUT1*) were found to be significantly associated with GY and its related traits (SSR and PN) under saline conditions, while two genes (*LOC_Os05g31730* and *LOC_Os01g23530*) were significantly associated with SSI, SSD, and SN at the seedling stage. Of course, further studies are needed to validate the functions of these genes, possibly by overexpression in transgenic plants or by CRISPR/Cas genome editing. Even these candidates are not causal genes, but the SNPs in the gene sequences are also suitable for marker-assisted breeding because of the high degree of linkage disequilibrium between them. Thus, after converting these linked SNPs into Kompetitive Allele Specific PCR (KASP) markers, marker-assisted breeding could be carried out to improve seedling development and yield production by deploying different yield and yield-related ST genes with seedling-stage-ST genes under saline field conditions. We identified seven accessions, including CX315, IRIS 313–11,571, IRIS 313–11,584, IRIS 313–11,652, IRIS 313–11,698, IRIS 313–9724, and IRIS 313–9725 that carry favorable haplotypes of the four genes (*LOC_Os02g49700*, *LOC_Os03g07480*, *LOC_Os03g28300*, and *LOC_Os04g52140*) that have high GY under saline stress conditions, and three accessions, including B255, CX250, and IRIS 313–10,275, that carry favorable haplotypes of two genes (*LOC_Os01g23530* and *LOC_Os05g31730*) with good ST at the seedling stage (Additional file 1: Table S7); all of these lines could be used as parents in rice ST breeding.

## Conclusion

ST characters varied widely in the rice association panel at both the seedling and reproductive stages under salt stress conditions. A GWAS utilizing high density SNPs within genes followed by functional annotation, GO analysis, and haplotype analysis was effective at detected candidate genes related to the investigated traits. We identified a total of 15 promising gene candidates, of which two are known to be involved in ST, three were previously cloned in relation to other traits but their roles in rice ST were not characterized, and another 10 novel genes that are potential candidates for rice ST. Seven high yield rice accessions carrying favorable haplotypes for the four genes significantly associated with GY under the saline stress conditions and three accessions with favorable haplotypes for the two genes with good ST at the seedling stage were identified. These promising candidate genes that we identified as being associated with rice ST are valuable resources for future functional characterization and marker-assisted breeding to improve rice grain yield and production in salinized paddy fields.

## Supplementary information

**Additional file 1: Table S1.** Rice accessions included in the ST association panel. **Table S2.** All identified marker-trait associations and the corresponding annotated genes. **Table S3.** Known genes involved in rice salt stress recorded in the QTARO and funricegenes databases. **Table S4.** The promising candidate genes for ST showed the same functional annotations and GO terms as genes known to be involved in salt stress. **Table S5.** ANOVA for haplotypes of 43 genes with the target traits. **Table S6.** ANOVA for haplotypes of the 15 promising genes with GY in each subgroup grown in two environments. **Table S7.** Well performing accessions carrying favorable haplotypes of four genes associated with GY and two genes associated with SSI and SSD

**Additional file 2: Figure S1A-I.** Manhattan and Q-Q plots of the GWAS for all measured traits

## Data Availability

The datasets used and/or analyzed during the current study are available from the corresponding author upon reasonable request.

## Abbreviations

3 K RGP: 3 K Rice genome project
ANOV: Analysis of variance.
FGN: filled grain number.
GAPIT: Genome Association and Prediction Integrated Tool.
GO: gene ontology.
GWAS: Genome-wide Association Study.
GY: grain yield per plot.
HD: heading date.
MAF: minor allele frequency.
QTL: quantitative trait loci.
SNP: single nucleotide polymorphism.
SSD: seedling survival days.
SSI: salt stress injury.
SSR: seed setting rate.
ST: salt tolerance.
SN: seedling number.
TGW: thousand-grain weight.
TPN: total panicle number.
TSN: total spikelet number per plot.
