# Supplementary material for: Identification of genes for salt tolerance and yield-related traits in rice plants grown hydroponically and under saline field conditions by genome-wide association study
Source: Rice (N Y). 2019 Dec 2;12:88. doi: 10.1186/s12284-019-0349-z (PMC6889114; doi:10.1186/s12284-019-0349-z)
Supplement: Supplementary file 2 — Additional file 2: Figure S1A-I. Manhattan and Q-Q plots of the GWAS for all measured traits [file 12284_2019_349_MOESM2_ESM.pptx]

## Slide 1
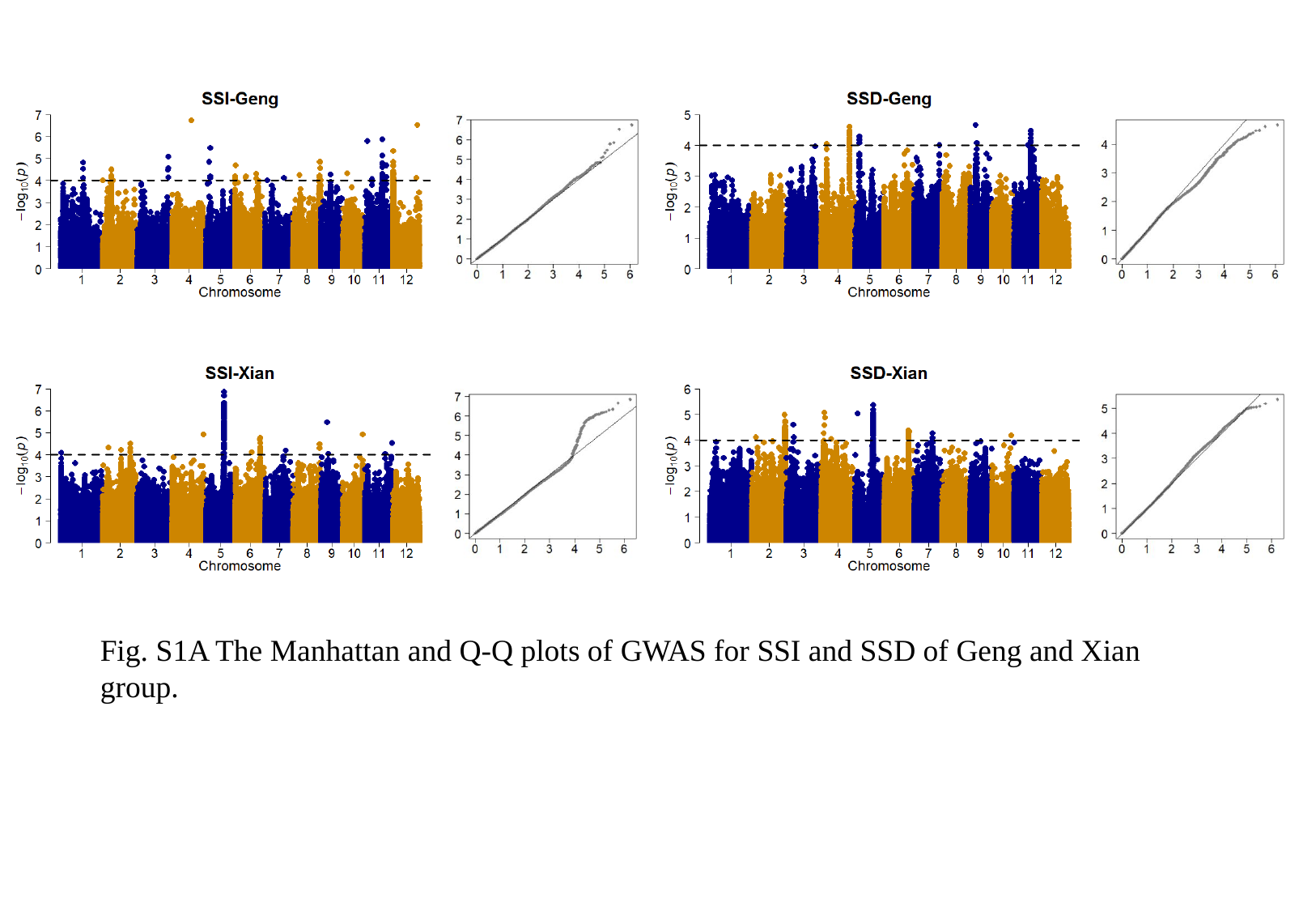

Fig. S1A The Manhattan and Q-Q plots of GWAS for SSI and SSD of Geng and Xian group.

## Slide 2
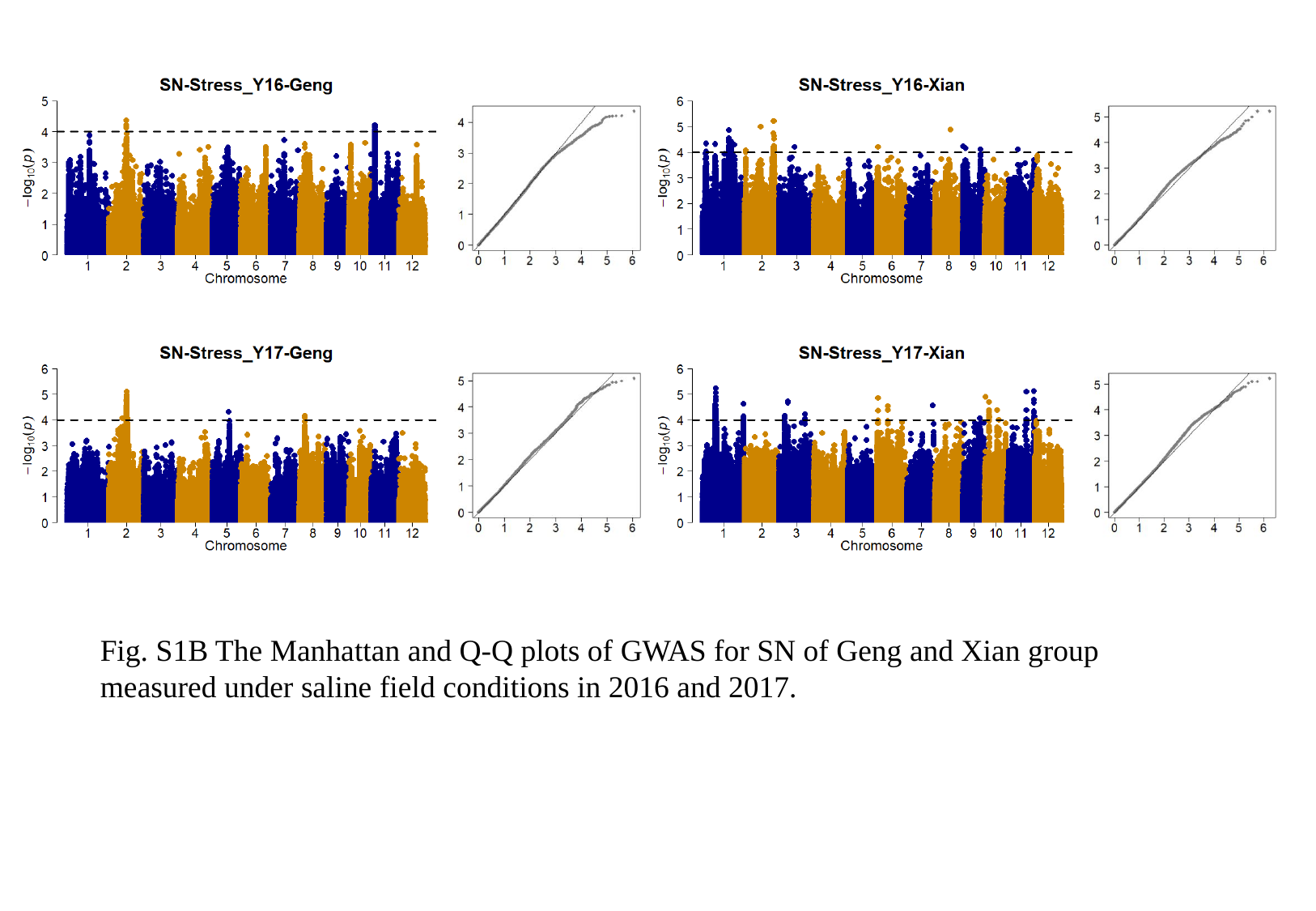

Fig. S1B The Manhattan and Q-Q plots of GWAS for SN of Geng and Xian group measured under saline field conditions in 2016 and 2017.

## Slide 3
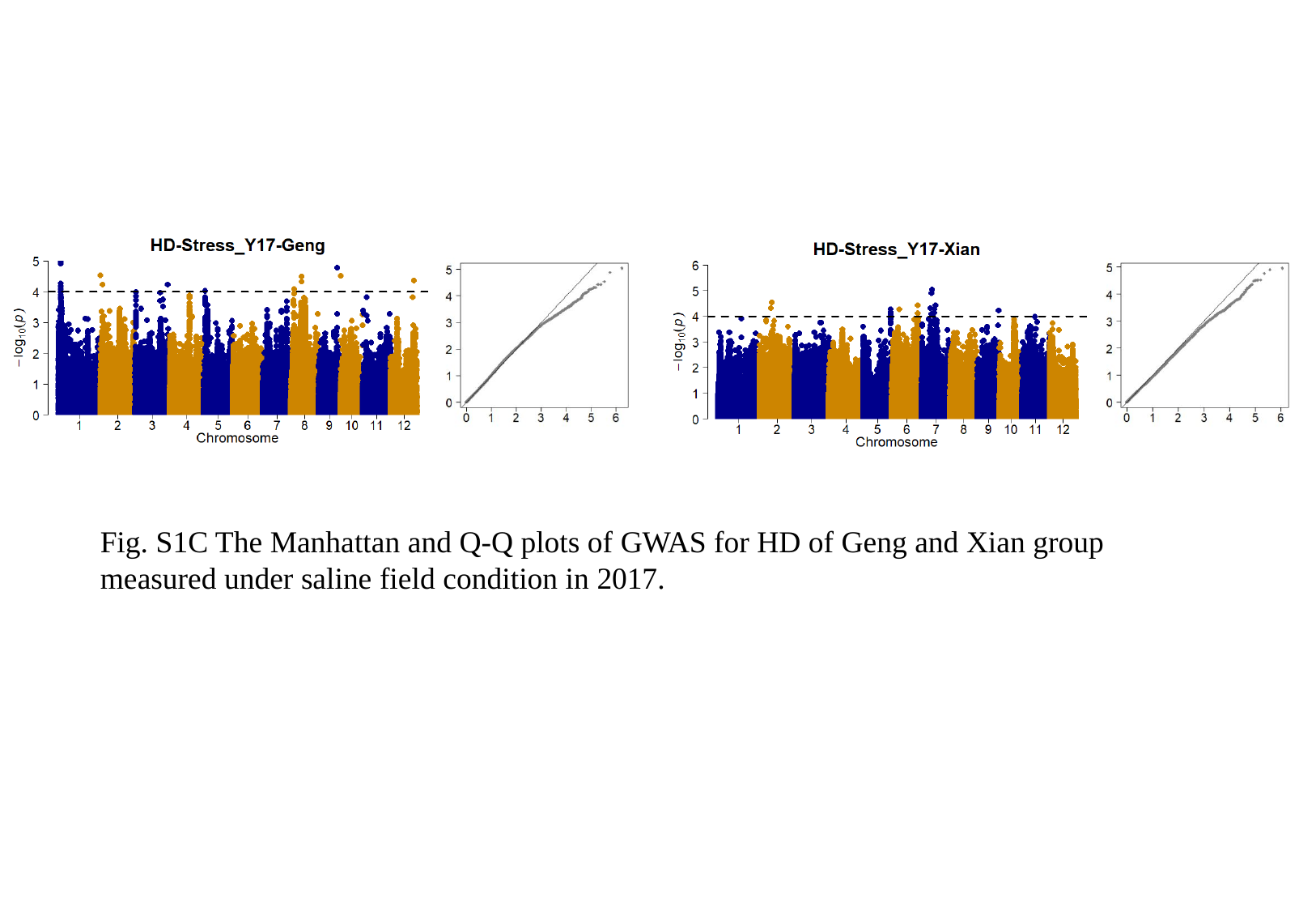

Fig. S1C The Manhattan and Q-Q plots of GWAS for HD of Geng and Xian group measured under saline field condition in 2017.

## Slide 4
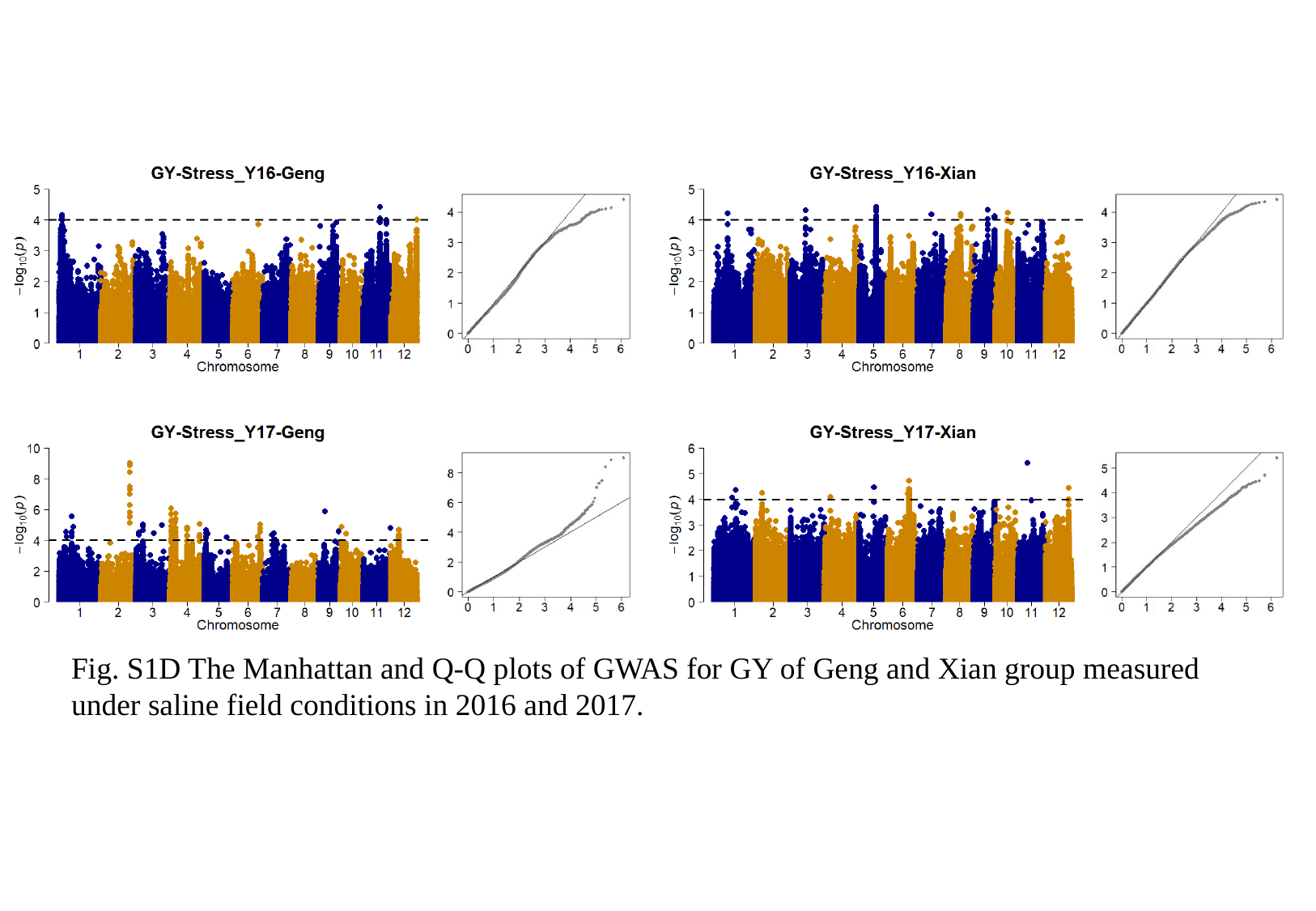

Fig. S1D The Manhattan and Q-Q plots of GWAS for GY of Geng and Xian group measured under saline field conditions in 2016 and 2017.

## Slide 5
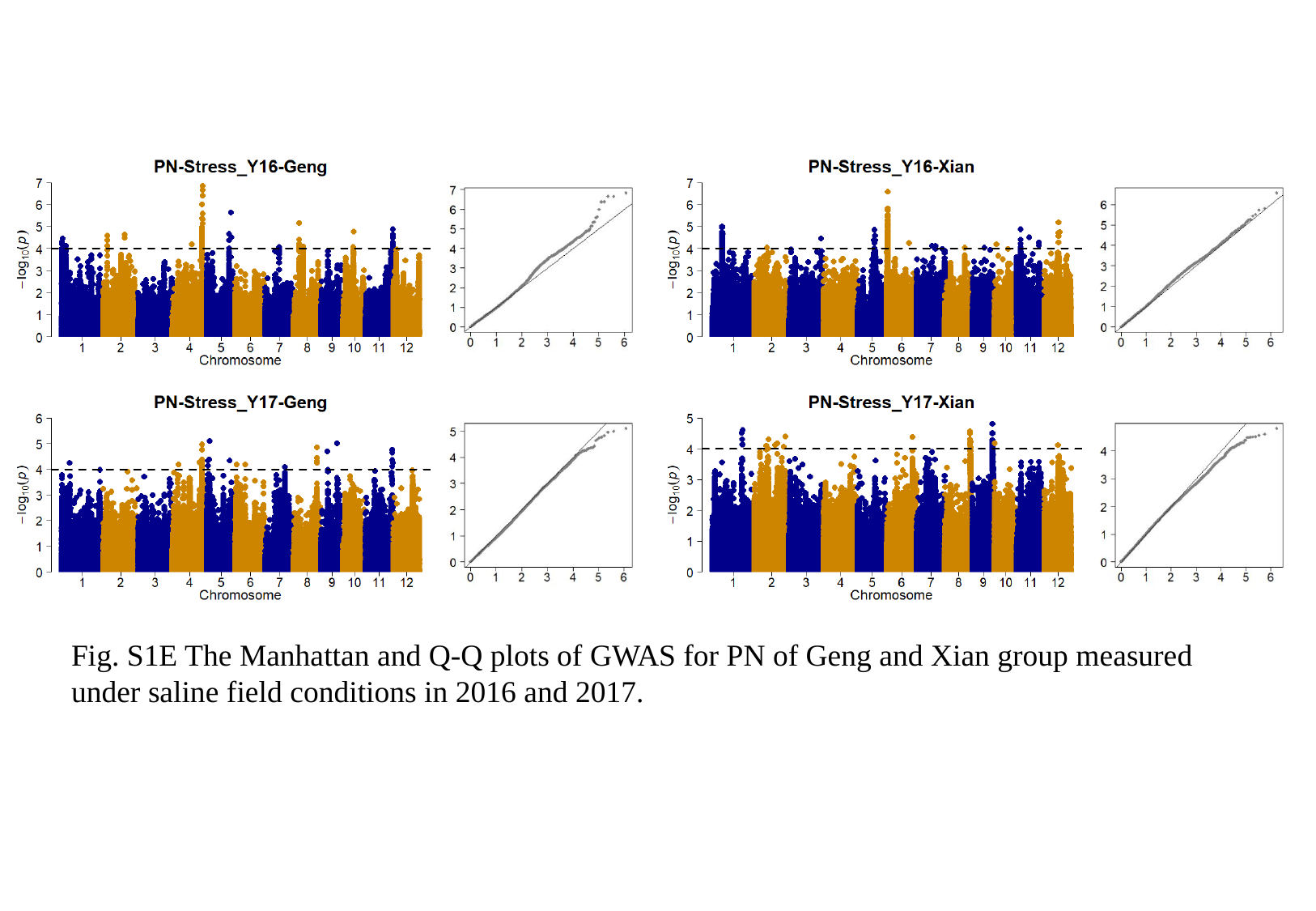

Fig. S1E The Manhattan and Q-Q plots of GWAS for PN of Geng and Xian group measured under saline field conditions in 2016 and 2017.

## Slide 6
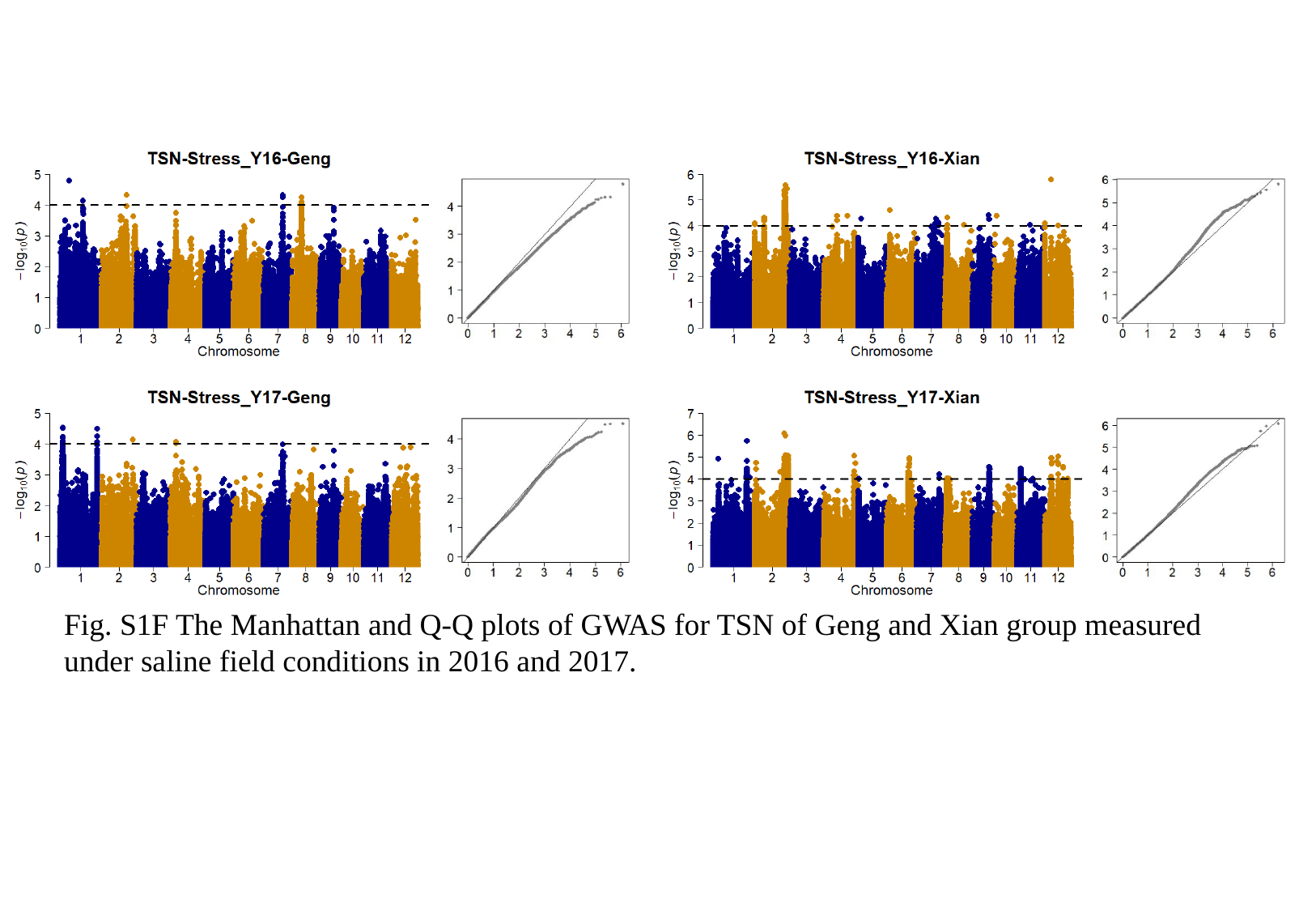

Fig. S1F The Manhattan and Q-Q plots of GWAS for TSN of Geng and Xian group measured under saline field conditions in 2016 and 2017.

## Slide 7
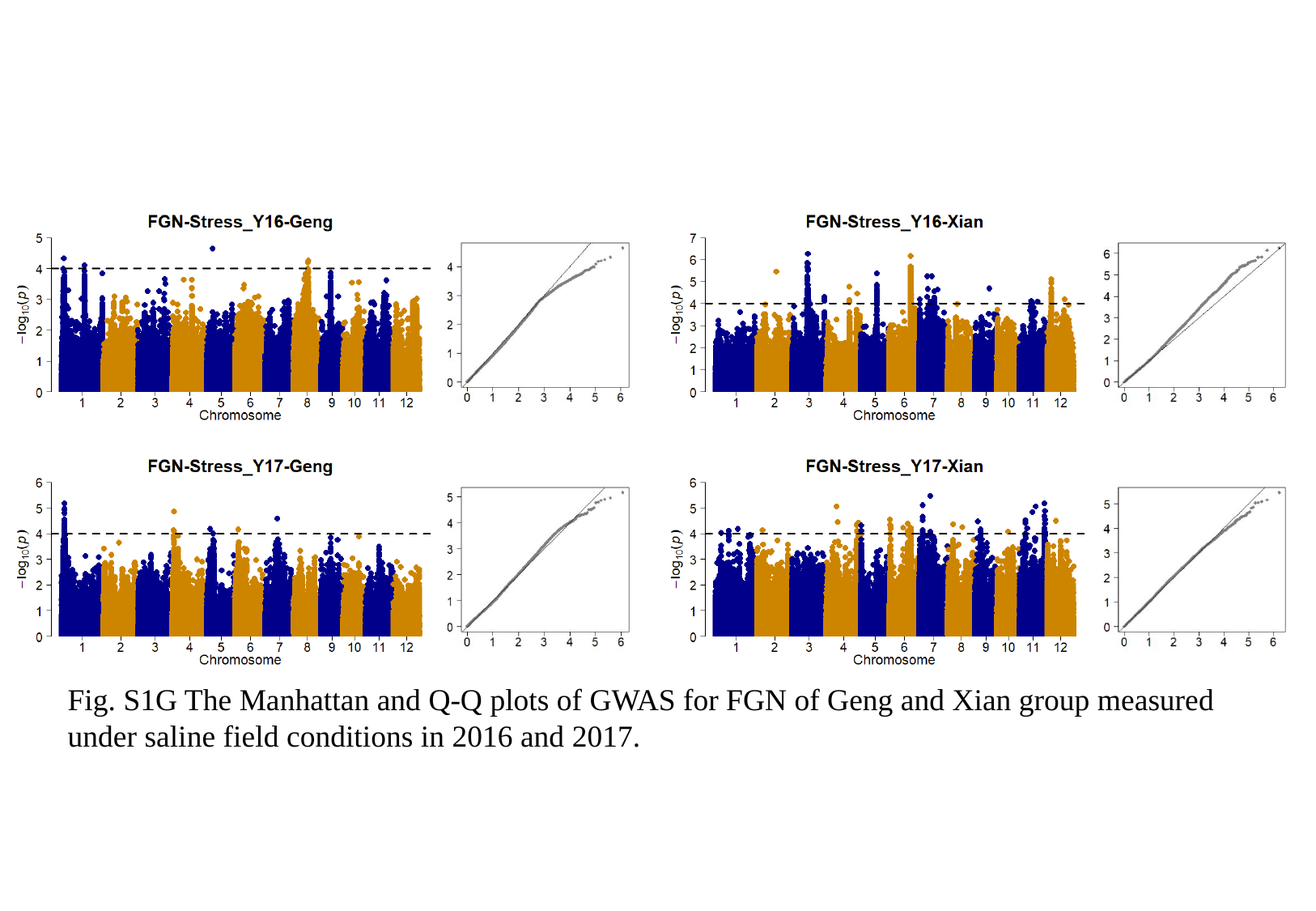

Fig. S1G The Manhattan and Q-Q plots of GWAS for FGN of Geng and Xian group measured under saline field conditions in 2016 and 2017.

## Slide 8
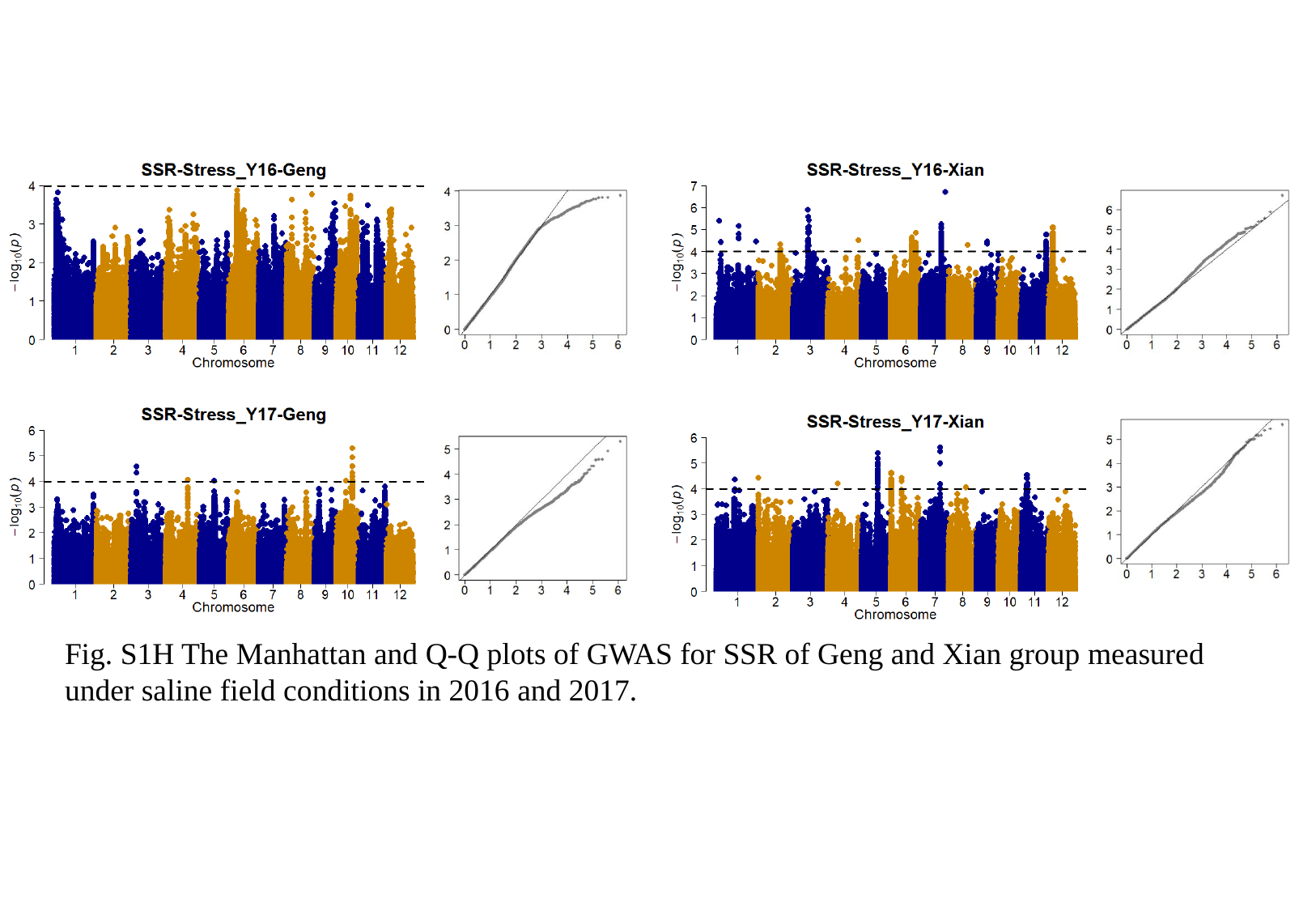

Fig. S1H The Manhattan and Q-Q plots of GWAS for SSR of Geng and Xian group measured under saline field conditions in 2016 and 2017.

## Slide 9
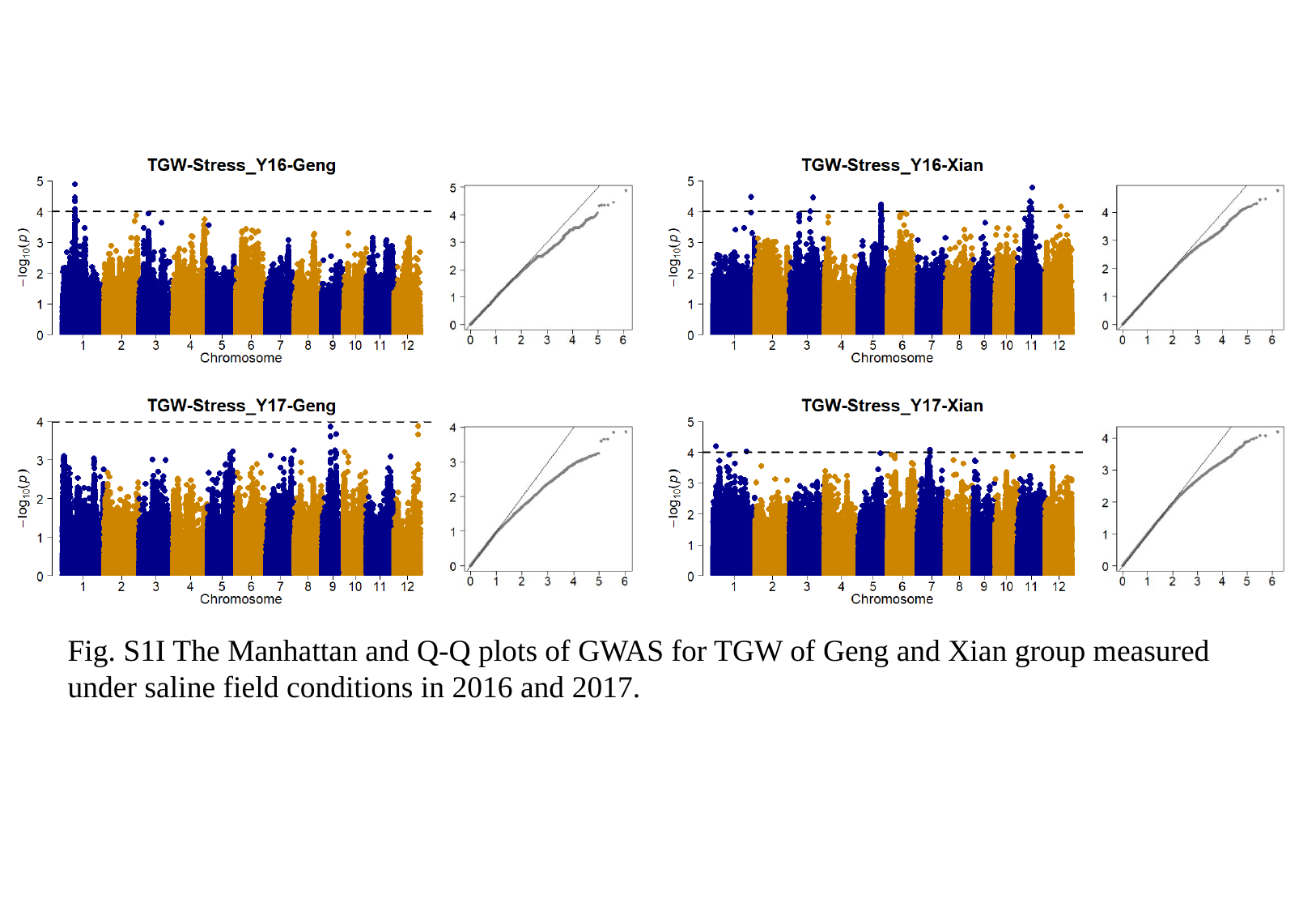

Fig. S1I The Manhattan and Q-Q plots of GWAS for TGW of Geng and Xian group measured under saline field conditions in 2016 and 2017.
